# Exploring Topics in Bibliometric Research Through Citation Networks and Semantic Analysis

**DOI:** 10.3389/frma.2021.742311

**Published:** 2021-09-24

**Authors:** Cristian Mejia, Mengjia Wu, Yi Zhang, Yuya Kajikawa

**Affiliations:** ^1^ Graduate School of Environment and Society, Tokyo Institute of Technology, Tokyo, Japan; ^2^ Faculty of Engineering and Information Technology, Australian Artificial Intelligence Institute, University of Technology Sydney, Ultimo, NSW, Australia; ^3^ Institute for Future Initiatives, The University of Tokyo, Tokyo, Japan

**Keywords:** bibliometrics, scientometrics, informetrics, topic extraction, strategic management, public health, sustainability

## Abstract

This article surveys topic distributions of the academic literature that employs the terms bibliometrics, scientometrics, and informetrics. This exploration allows informing on the adoption of those terms and publication patterns of the authors acknowledging their work to be part of bibliometric research. We retrieved 20,268 articles related to bibliometrics and applied methodologies that exploit various features of the dataset to surface different topic representations. Across them, we observe major trends including discussions on theory, regional publication patterns, databases, and tools. There is a great increase in the application of bibliometrics as science mapping and decision-making tools in management, public health, sustainability, and medical fields. It is also observed that the term bibliometrics has reached an overall generality, while the terms scientometrics and informetrics may be more accurate in representing the core of bibliometric research as understood by the information and library science field. This article contributes by providing multiple snapshots of a field that has grown too quickly beyond the confines of library science.

## Introduction

Bibliometric methodologies are considered useful as supporting tools for decision-making in setting research priorities, tracking the evolution of science and technology, funding allocation, and rewarding scientific excellence, among others. Given their versatility, these methods have quickly spread beyond the information and library science domain from where they initiated. Part of this spread is due to an abundance of data and ease of accessibility. Also, due to the increase of processing and analytical tools with a varying range of complexity, making current bibliometrics reachable to scientists and practitioners at any level of expertise (Zuccala, 2016; Rousseau and Rousseau, 2017).

The spread of bibliometrics beyond library science is well-documented (Egghe, 2005a). Some authors, go as far as to raising concerns of its uncontrollable use and expanse, particularly when there is no knowledge of good practices among authors who execute those methods in distant fields (Johnson, 2011; González-Alcaide, 2021). Nevertheless, when executed properly bibliometric methods offer an abundance of benefits to other disciplines and it cannot be expected to be contained. In this direction, bibliometricians are left with the task of documented the development of the field and explaining its characteristics as it evolves.

Studies that focus on the spread and adoption of bibliometrics research have been concerned with establishing boundaries and definitions among information metrics (Milojević and Leydesdorff, 2013); surveying the development of the field through the lenses of information and library science expertise (Schoepflin and Glänzel, 2001); or, establishing comparisons between bibliometrics in library science versus other fields (Jonkers and Derrick, 2012). So far, most studies have neglected the academic landscape of the field from a point of view of its intrinsic bibliographic characteristics. This article is aiming at closing that gap. Specifically, this article aims to bring topical representations of the literature that explicitly acknowledge being part of bibliometric research by surfacing topics from patterns in the citation network of the field, hierarchical semantic relationships, and a combination of both. In doing so, we provide topical representations that are not forced into predefined classifications but that respond to the organic development of the field.

The rest of the article is structured as follows. We present a brief introduction of the definitions and history of bibliometrics research and the previous efforts on mapping the field. Then, three methods are introduced, and results displayed. We conclude by discussing the characteristics of the topics found, the connection and differences between methods, and the challenges of topic detection.

## Previous Literature

### An Overview of Bibliometric Research

Activities that could be considered as part of what we know today as bibliometrics have been traced back to the 12^th^ century with Hebrew citation indexes (Weinberg, 1997), while the usage of publication counts has been observed in legal documents of 1817 (Shapiro, 1992). More formally, as an academic work, Campbell proposes the use of statistics for cataloging and indexing literature of mathematics and natural sciences and puts efforts towards a bibliographical reform (Campbell, 1896). Godin adds to the discussions of the origins of bibliometrics that around those years and the early 1900s psychologists started to collect statistics of the discipline, to establish some sort of indicators for the measure and advancement of psychology work (Godin, 2006).

All the previous examples come from a time when such practices did not have a name. However, as the volume of those activities increased, researchers started to name it. According to Pritchard, they were labeled *statistical bibliography* by E. Wyndham Hulme during lectures given in Cambridge in 1922 (Pritchard, 1969). The term statistical bibliography was later defined by Raisig in 1962 as the “assembling and interpretation of statistics relating to books and periodicals … ” (Raisig, 1962). Another term used to describe these studies is *librametrics* coined by Ranganathan in 1948 (Sen, 2015). The usage of *statistical bibliography* and *librametrics* as terms describing bibliometric research is currently largely discontinued.

The first instance of the term *bibliometrics* was found as its French equivalent hidden in a section titled “Le Livre et la Mesure. Bibliometrie.” within the book Traité de Documentation by Paul Otlet (Otlet, 1934). However, it is argued [(Sengupta, 1992; Hood and Wilson, 2001)] that the term reached worldwide spread thanks to the work of Pritchard in 1969 (Pritchard, 1969). This is the same year when the term *Scientometrics* was coined by Nalimov and Mulchenko in its Russian equivalent *Naukometriya* (Nalimov and Mulchenko, 1969). The term Scientometrics would gain popularity after the journal of the same name was founded in 1978. Finally, *Informetrics* has a more recent origin; proposed by Otto Nacke in 1979 (Brookes, 1990; Tague-Sutcliffe, 1992). In-depth details of the history of bibliometrics research can be seen in Thelwall (2008) and Dutta (2014).

The terms bibliometrics, scientometrics, and informetrics have continued in use until today. As they grew independently there were some efforts to establish clear boundaries (Bonitz, 1982). However, the research community seems to have accepted the great overlap among them and agreed on their interchangeable use in some cases. Informetrics is the one with the largest scope referring to the study of quantitative aspects of information in any form, including and beyond the academic community and academic outputs (Jacobs, 2010). Hence, informetric studies cover both bibliometrics and scientometrics.

Bibliometrics refers to “the application of mathematics and statistical methods to books and other forms of written communication” (Pritchard, 1969). On the other hand, scientometrics refers to “all quantitative aspects of science and scientific research” (Sengupta, 1992). According to these definitions, the overlap between the metrics becomes apparent when dealing with the production and analysis of academic literature, being the written representation of academic outputs. An example of scientometric studies not about bibliometrics can be those measuring the third function of the University (a.k.a. contribution to society) or the measurement of university-industry collaboration in the cases where no written output is available (or feasible). On the contrary, a statistical analysis of literary production of fictional works by writers not affiliated to academia, may be labeled as a bibliometric study but not as a scientometrics one.

As the three metrics evolve, different authors have developed nuanced definitions for each of them. Some articles reviewing bibliometric definitions are Chellappandi and Vijayakumar (2018) and Hlavcheva et al. (2019). While comprehensive overviews of the field can be found with Glänzel (2003) and Rousseau et al. (2018).

### Bibliometric Studies on Bibliometrics

Not surprisingly, researchers have applied bibliometric methods to understand the field of bibliometrics. These meta-studies rely on the systematic extraction of bibliometric-related research from academic articles databases. Then, a battery of methods ranging in complexity is applied to those datasets to understand key players (authors, journals, institutions, countries, etc.) or topical trends within the field. The approach to capture bibliometric research has varied depending on the scope of the articles, from analyzing the whole field of library and information science, or leading journals of the field, to topical searches based on keywords. In this section, we present those works.

Aiming to present a historical survey on bibliometrics, scientometrics, and informetrics Hood and Wilson (2001) analyzed articles classified into the “information science” category of the DIALOG database obtaining 4,697 records by the year 2000. The overlap between the three terms was surfaced with a prevalence of usage of the term “bibliometrics” over the others. They also noted the appearance of related terms like Technometrics, netometrics, webometrics, and Cybermetrics.

Another approach to capture trends in bibliometric research is to study the articles published by leading journals in the field. In this direction, Schoepflin and Glanzel collected articles published in 1980, 1989, and 1997 in the journal *Scientometrics* and manually classified the retrieved records into any of six categories: “1. Bibliometric theory, mathematical models and formalization of bibliometric laws, 2. Case studies and empirical papers, 3. Methodological papers including applications, 4. Indicator engineering and data presentation, 5. Sociological approach to bibliometrics, sociology of science, 6. Science policy, science management, and general or technical discussions” (Schoepflin and Glänzel, 2001). These categories were set by the authors to track variations over time, revealing a balanced distribution for the first timeframe, while case-study papers becoming dominant in the most recent timeframe of the study. A similar dataset with articles from *Scientometrics* was used by Schubert to gain insights on co-authors, citation patterns, and regional trends (Schubert, 2002), although not specifying any topical categorization other than the same reported by Schoepflin and Glanzel.

While *Scientometrics* is considered the leading journal on bibliometric research, other journals have also played a major role. Janssens et al. (2006) also included the *Information Processing and Management Journal*, *the Journal of the American Society for Information Science and Technology, the Journal of Documentation*, and the *Journal of Information Science* in an effort for mapping the field of library and information science, with this being one of the first meta-studies applying an automatic topic detection method extracting topics from clusters of keywords. The topics identified were bibliometrics (2 clusters), patent analysis, information retrieval, webometric, and social studies and applications. In a similar study, Milojević and Leydesdorff (2013) applied bibliometric methods to articles from *Scientometrics*, the *Journal of Informetrics*, and *the Journal of the American Society for Information Science and Technology* to reveal key authors and profile each journal based on their most salient keywords. They found that bibliometrics, scientometrics, informetrics, and webometrics are developing a distinctive and cohesive vocabulary that grows faster in relation to other topics within information science.

The other prevalent approach for data acquisition in these meta-studies is the use of keywords to perform topical searches in bibliographic databases. In reviewing trends on information-metrics research, Bar-Ilan (2008) develops a comprehensive query listing a variety of terms related to bibliometric methods. Data were extracted from the Web of Science (WoS), Scopus, Google Scholar, and other databases for the years 2000–2006, leading to 598 articles after filtering. It was found that traditional topics like citation analysis, impact factor, and h-index research continue on the rise, but also newer ones like webometrics, mapping and visualization, and open access are being introduced as recurrent topics in bibliometrics. Other articles apply a similar approach by using different keyword combinations resulting in overlapped datasets from where generic statistical summaries are computed (e.g. (Mooghali et al., 2011; Ellegaard and Wallin, 2015)).

Is until more recently that we start observing meta-studies applying network analysis methodologies to obtain insights on the similarities and differences between the metrics. Siluo and Qingli (2017) retrieved 6,688 articles matching the keywords bibliometrics, scientometrics, and informetrics from the WoS, and studied the co-author cooperation network, and co-word network separately for each metric to understand their overlaps and divergences. Their article confirms a great topical overlap between the three, with informetrics surfacing keywords related to mathematical models.

With a dataset of 23,296 articles obtained from a longer list of search terms, Maltseva and Batagelj (2020) apply citation analysis to uncover evolutionary pathways, or citations chains, among researchers of bibliometrics. They are particularly interested in uncovering collaboration patterns finding that the number of published articles on bibliometrics doubles every 8 years and that collaborative articles featuring three or more authors is increasing compared to the decreasing trend of single-authored papers.

Following a keyword-based approach for data extraction but with the scope of finding differences between bibliometric research within and outside information and library science we found the work of Jonkers and Derrick (2012) who studied 3,852 bibliometric articles published between 1991 and 2010 and compared citations and author from articles published in library science journals and articles in other journals. They found that bibliometric research in library science received not statistically significant more citations than the one in other fields. This type of comparative analysis has continued with Larivière (2012) and Ellegaard and Wallin (2015) and Ellegaard (2018) each using comparable data and methods although pursuing different levels of granularity in their comparisons. They all coincide with the great spread of bibliometrics beyond library science. Most notably, González-Alcaide (2021) do the comparison considering the author collaboration networks and different domain levels including library science, social science, life science and medicine, technology, physical science, multidisciplinary science, and arts and humanities as categories of evaluation, noting few collaboration ties to the core of bibliometric research and dispersed teams working independently.

Finally, we circle up this section by mentioning the work of Li et al. (2019) where the authors collected articles under the category of information and library science in the WoS to map the field by covering articles from 1989 to 2018. From our records, this is the meta-study (bibliometrics on bibliometrics) with the largest dataset covering 88,304 articles. It can be seen as an update to the work of Hood and Wilson (2001), which opens this section. They reveal that library and information science is divided into 8 clusters: information retrieval theories, social media, the impact of information systems on organizational management, key elements of information system, information behavior, bibliometrics and webometrics, information retrieval technology, and scientific evaluation. Hence, we observe that the field of information and library science cannot be considered anymore as a proxy for bibliometrics research. And that bibliometrics is a distinct cluster part of it.

The present article is also a meta-study on bibliometrics. Hence, we would like to point the differences in the articles previously discussed. We share a data collection strategy similar to that of Siluo and Qingli (2017). However, this article is not interested in elucidating differences between the three metrics, on the contrary, we analyze them as a consolidated research corpus. Additionally, while we attempt to review the major topical trends on bibliometrics we do not sort the corpora into predetermined classification schemas like in Schoepflin and Glänzel (2001) or derive an expert-based outline of topics as in Bar-Ilan (2008). Here, we show topics derived from the network structure of academic articles, which is in itself a reflection on how the topics have evolved “naturally” within the academic community. Therefore, our expected contribution is a snapshot of the major topical trends as seen from academic literature that acknowledge an explicit association to bibliometric research.

## Data and Methods

### Data

The WoS Core Collection was used as the source for bibliographic data. WoS was developed by the Institute of Scientific Information and is currently maintained by Clarivate Analytics. The core collection includes the Science Citation Index Expanded, the Social Sciences Citation Index, the Arts & Humanities Citation Index, the Emerging Sources Citation Index, the Book Citation Index, and the Conference Proceedings Citation Index, hence spanning across multiple disciplines and document types. To obtain documents explicitly employing the concerning terms we performed a topical search with the query TS = “bibliometr*” OR “scientometr*” OR “informetr*”. In the query, the asterisk serves as a truncation symbol to accommodate variations of the queried term (e.g., bibliometric, bibliometrics, bibliometrician). A topical search retrieves records matching the query in the title, abstract, or keywords. No time constraints were placed searching for records in all years available in the database. Data were retrieved on March 20, 2021, obtaining 20,268 records. This dataset is composed of 70.4% of journal articles, 13% of proceeding papers, 12.2% of reviews, and the remaining 4.4% of other types including editorial material and book chapters. All types of records were included in this research, and we refer to them simply as “articles” in the remainder of this paper. The full list of articles including the database’s article ID, document object identifier, and a label indicating whether they matched any of the queried terms is offered as supplementary material.

### Methods

**FIGURE 1:**
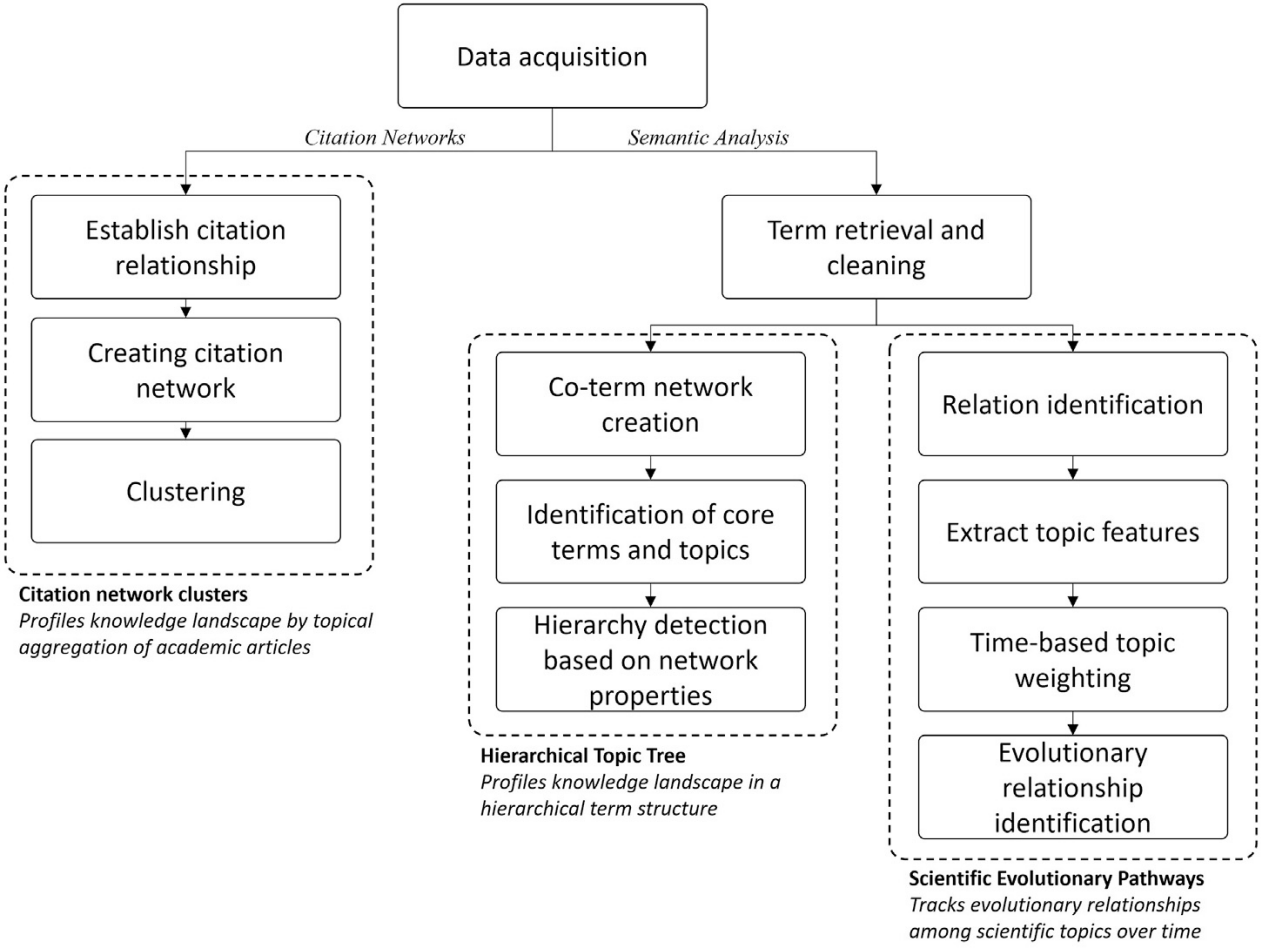
Research framework.

We applied three methodologies to uncover the topical trends in the bibliometrics dataset. These are applied independently and are selected to exploit different features of the dataset. An overview of the methods is shown in Figure 1. First, we extract topics from the direct citation network of publications. Next, we built a hierarchical topic tree based on the structure of a co-occurrence network of terms. Finally, a method combining both, term analysis and citation analysis, to observe scientific evolutionary pathways. These methodologies are established, and details of the implementation and exemplary case studies can be found elsewhere. In the following, we present a summary of their basic construction and properties.

The first method is the construction of topics through the clustering of a direct citation network. These networks are built by simply establishing a linkage between two academic articles when one cites the other (de Solla Price, 1965). Direct citation networks are known to surface research field taxonomies (Klavans and Boyack, 2017) and help in identifying research fronts (Shibata et al., 2011). They work best when the time window of analysis is long, as it is for bibliometrics research. Therefore, it is the approach selected in this article, although other types of networks exist that could help for different objectives. For instance, co-citation networks help identifying core and foundational research (Small, 1973) and bibliographic coupling networks are useful when the time frame is short (Kessler, 1963).

Identifying topics from a citation network works as follows. Articles in the dataset are treated as nodes. A link is drawn between a pair of nodes when one cites the other, thus generating a citation network. In a citation network from academic articles, it is expected for some groups of nodes to have denser connections when compared to other groups of nodes. An optimal partition of the network is achieved when the link density is higher at the intra-cluster level than the inter-cluster level. To reach an optimal partition, it is necessary to group nodes in a manner that maximizes the modularity of the network, which is a measure of the strength of the division of a network into communities (Clauset et al., 2004; Fortunato, 2010). The Louvain method (Blondel et al., 2008) is commonly applied to partition citation networks of academic articles and is the one used in this article. This algorithm is known to be computationally efficient when partitioning large networks (Šubelj et al., 2015). Once the clusters are obtained, we calculated summary statistics of the publication years and citations received by their articles. Clusters were named by the authors based on an assessment of the titles of the most connected articles, the cluster most frequent keywords, or relevant metadata like journal names, countries, or authors.

The second method is a hierarchical topic tree (HTT). This method was developed to identify topic hierarchies by incorporating density peak searching and overlapping community allocation algorithms with a co-term network. Specifically, the HTT approach creates a way to re-organize a co-term network into a tree structure. It first computes pairwise shortest topological distances of all nodes in the network and assigns each node a local density calculated by its K nearest neighbor according to the formula in (Wu and Zhang, 2020). Then, nodes with the highest density among their corresponding K neighbors are identified as density peak nodes and constitute roots in the ultimate tree. The rest nodes in the network are assigned to their closest root to form communities. Then, this process is recursively applied to each community to identify child density peak nodes (i.e., leaves) at different hierarchies. A link between two leaves on the same branch indicates the strength of their closeness, calculated by the topological distance between their related nodes in the network.

The third method is scientific evolutionary pathways (SEP). It is inspired by the theory of technological recombination (Fleming, 2001), the SEP approach stands on the assumption that cumulative changes of existing knowledge will result in scientific evolution, and such knowledge can be represented by topics–a collection of research articles touching similar research contents. This approach exploits streaming data analytics to identify the predecessor-descendant relationships between research topics by measuring the semantic similarity between new research articles and existing topics and deciding which existing topic the new articles belong to and whether cumulative changes occur or not.

### Implementation

Bibliographic data including the full record and cited references were exported as tab-delimited files from the database website^1^. This dataset was then processed with the statistical software R version 3.6.3 (R Core Team. R, 2019). The package igraph version 1.2.5 (Csardi and Nepusz, 2006) was used to create the network and obtain clusters, and the package tm version 0.7.7 (Feinerer et al., 2008) was used for text processing. The citation network was visualized by applying the large graph layout (LGL) (Adai et al., 2004). The selection of LGL was based on computational efficiency and the selection of it over other layouts has no impact on the results of this research. These packages are open source, and their code is available in their respective GitHub repositories.

With the same dataset but the raw records in the tab-delimited files, we ran the HTT approach in its Python platform and visualized the results via the tree layout developed by Vega^2^, and the SEP approach was developed via Python as well but its network was created with the aid of Gephi (Bastian et al., 2021) and the nodes were colored by applying the included community detection function based on modularity maximization (Newman, 2006).

## Results

**FIGURE 2:**
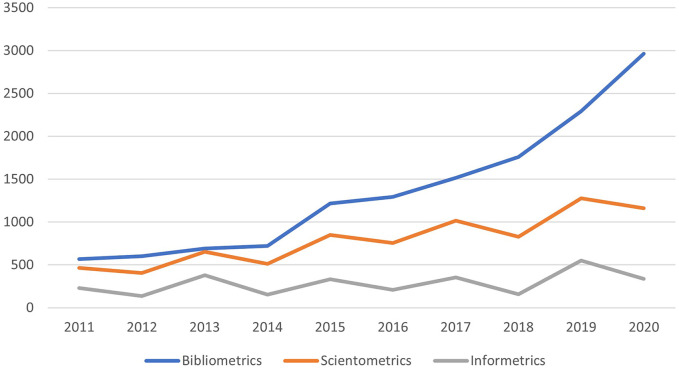
Number of articles published per year.

Academic articles on bibliometrics were retrieved from a bibliographic database resulting in 20,268 articles published between 1969 and 2021. The earliest records correspond to the seminal work of Pritchard (Pritchard, 1969) where the term “bibliometrics” is formally introduced in English written research, and the work of Fairthorne (1969) who surfaces the presence of the Bradford, Zipf, and Mandelbrot distributions when quantifying academic literature. As seen in Figure 2, the three terms continue in use, with bibliometrics being the most popular in the academic community in terms of publications per year, reaching 2,966 publications in 2020. On the other hand, informetrics, despite being conceptually the broadest, is the less observed with an average number of publications of 285 articles per year over the past decade. It is also observed a small boost of publications on scientometrics and informetrics every 2 years, corresponding to years in which the International Conference of Scientometrics and Informetrics, the leading conference in the field, takes place.

**FIGURE 3:**
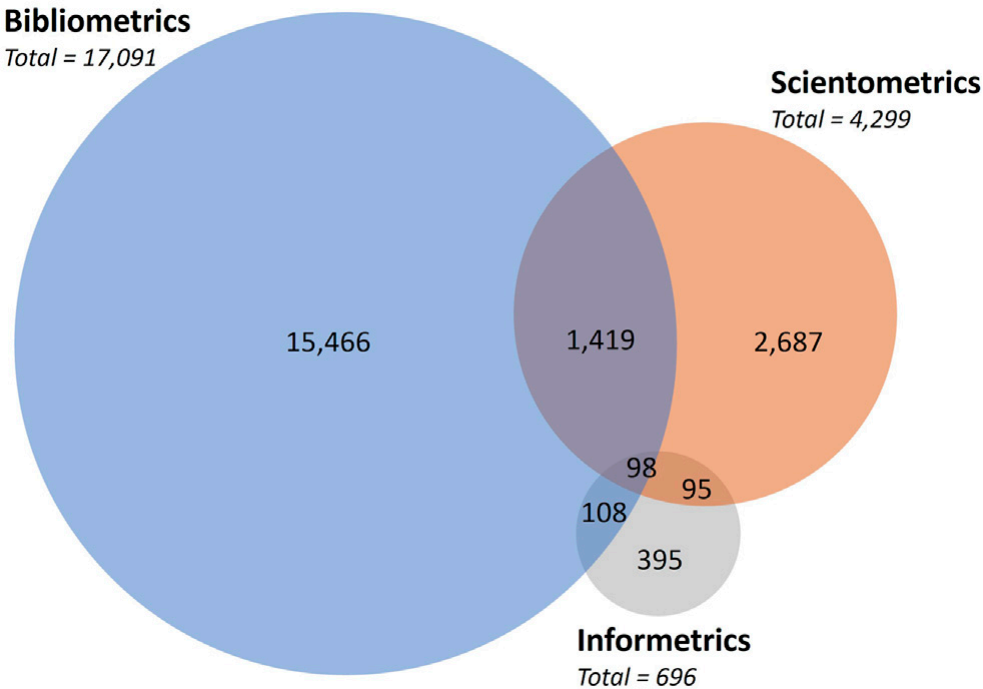
Number of articles containing the keywords “bibliometrics”, “scientometrics” or “informetrics” in their title, abstract, or indexed keywords.

Authors in this field tend to identify their works with either of the terms. However, some works contain a combination of these keywords in the title, abstract, or keywords. This overlap is shown in Figure 3. The three terms are mentioned simultaneously in 98 articles which are mostly studies on information metrics, or meta-studies on the field. The largest overlaps occur with bibliometrics and scientometrics, having 1,469 articles mentioning both terms.

### Topical Distribution From a Citation Network

**FIGURE 4:**
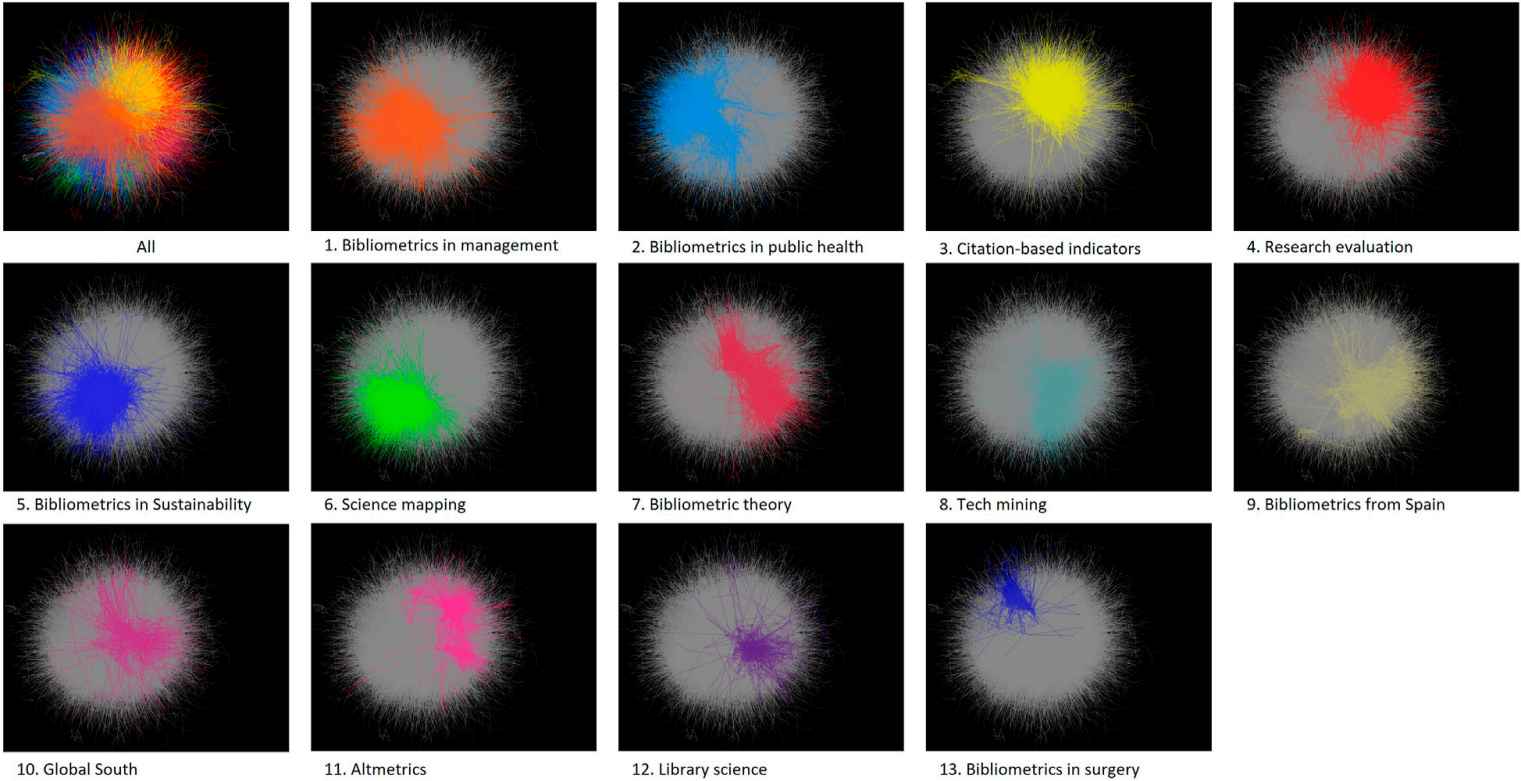
Citation network of bibliometric research showing the relative position of the 13 clusters identified.

To understand major trends within the combined dataset we extracted clusters from the direct citation network of the articles. The largest connected component consists of 17,150 articles (85% of the dataset) from where 13 clusters were identified. An additional cluster titled “others” is used to aggregate clusters of neglectable size. The remaining 15% of disconnected articles were reprocessed in an attempt to find other disconnected clusters but none were found. Figure 4 shows the network and its clusters. In this visualization, only edges are plotted. Clusters were named based on the contents of their most cited works, most frequent keywords, or by bibliographic characteristics like subject fields or dominant countries. These names serve only as an approximation of their contents and are expected to contain a plurality of related subtopics within them, hence, they are provided as guidance. The purpose of this figure is to offer an indication of the relative size and relative position of the clusters in the network. Clusters located in a similar position may signal a topical overlap, as is observed for clusters 2 and 13 of bibliometrics in public health and surgery, respectively. Also, it can be expected that the cluster of research evaluation and the one of citation-based indicators have a common knowledge base given that citation metrics are sometimes used in the context of research evaluation. This link is confirmed by observing that clusters 3 and 4 are located in a similar position in the network. A quantitative summary of the clusters is offered in Table 1.

**TABLE 1:** Summary of clusters in the citation network including the share of articles containing the terms bibliometrics (B), scientometrics (S), and informetrics (I).

*n*	Cluster name	Articles	Publication year^a^			Times cited^b^			B %	S %	I %
			Min	Mean	Med	Mean	Med	Max			
1	Bibliometrics in management	2,306	1969	2017.6	2019	17.0	3	1871	15.2	4.8	2.6
2	Bibliometrics in public health	1914	1981	2016.4	2018	10.0	4	353	12.2	7.3	1.0
3	Citation-based indicators	1856	1982	2015.0	2016	17.4	5	654	10.5	12.7	10.6
4	Research evaluation	1831	1979	2012.9	2015	15.7	5	465	10.8	10.7	5.3
5	Bibliometrics in Sustainability	1756	1980	2015.4	2017	15.3	6	543	11.4	7.6	2.2
6	Science mapping	1,490	1983	2017.3	2019	14.5	3	2039	8.0	13.2	4.5
7	Bibliometric theory	1,316	1969	2007.6	2011	15.2	5	977	6.0	10.3	45.8
8	Tech mining	1,308	1980	2013.2	2015	21.0	6	2,281	7.2	9.3	6.5
9	Bibliometrics from Spain	834	1975	2011.9	2014	10.4	4	300	5.3	2.9	0.2
10	Global South	707	1980	2009.8	2013	9.9	4	350	3.2	8.6	5.7
11	Altmetrics	558	1985	2014.8	2017	19.2	4	556	3.0	3.8	9.6
12	Library science	487	1972	2011.3	2014	11.5	4	289	2.7	3.7	4.6
13	Bibliometrics in surgery	371	1991	2015.8	2017	14.5	7	537	2.5	0.7	0.0
14	Others	416	1979	2015.9	2018	12.8	4	379	2.1	4.3	1.4
—	Total	17,150	—	—	—	—	—	—	100.0	100.0	100.0

a The most recent publication year (Max) by articles in each cluster is 2021.

b The minimum citations received (Min) by articles in each cluster is 0.

Clusters are sorted from the largest based on the number of articles they aggregate. The largest one labeled bibliometrics in management contains bibliometric studies on business, organizational theory, marketing, innovation, and other topics surrounding the field of management. For instance, studies surveying the application of bibliometric methods in management research (Zupic et al., 2015), or studies tracking the evolution of the field through quantitative methods (Ramos-Rodríguez and Ruíz-Navarro, 2004). Its most cited article deals with a study of big data analytics for business intelligence (Chen et al., 2012). Case studies at the journal level are common, including overviews of the European Journal of Operational Research (Laengle et al., 2017), European Journal of Marketing (Martinez-Lopez et al., 2018), or the Journal of Business Research (Merigo et al., 2015) among the most cited. This cluster is also the youngest in terms of the average and median publication year of its articles.

The second cluster contains bibliometric studies related to public health, surgery, and medicine. Research in this cluster analyzes top research in neurosurgery (Ponce and Lozano, 2010), radiology (Pagni et al., 2014), and others. Also bibliometric studies regarding specific diseases like a survey on the Middle East respiratory syndrome (Zyoud, 2016) and lung cancer (Aggarwal et al., 2016) among others.

Cluster 3 on citation-based indicators is the largest cluster within the field of information and library science. This cluster includes discussions of the meaning and measure of citations in academic research (Bornmann and Daniel, 2008); and how different data sources influence the results of such indicators (Meho and Yang, 2007; Harzing, 2016). And several critics to established indicators like the h-index and point to methods towards more useful metrics in the evaluation of scientific outputs (Leydesdorff et al., 2011; Waltman et al., 2011; Waltman and Van Eck, 2012).

While cluster 3 focuses on the construction of the indicators, cluster 4 deals with the applicability and implication of the usage of those indicators in several contexts. For instance, top-cited research in this cluster targets bibliometrics as a monitoring tool for research performance (Moed et al., 1985; Nederhof, 2006), most of these approaches are oriented towards the evaluation of performance in the social sciences and humanities (Hicks, 1999; Archambault et al., 2006). Additionally, it is explored the applicability of bibliometric methods as substitutive of peer review during the publication process (Rinia et al., 1998) or funding allocation (Abramo et al., 2009). This is the cluster with the second largest proportion of articles containing the keywords scientometrics and informetrics.

Cluster 5 pertains to bibliometric studies on environmental science, ecology, energy and fuels, climate change, and other topics around sustainability. Among the most cited articles in this cluster, we find the study of the collaboration network of scientists on the topic of resilience (Janssen et al., 2006), and a bibliometric study on tsunami research (Chiu and Ho, 2007), Others include studies on climate change (Li et al., 2011), a study of high impact articles in water resources (Chuang et al., 2011), and aerosol research (Xie et al., 2008).

Cluster 6 focuses on science mapping tools and studies applying such tools. Science mapping, also known as academic landscape or bibliometric cartography, allows users to quickly obtain insight from academic fields by plotting bibliographic data into visual representations (Cobo et al., 2011; Chen, 2017). Common methods include co-word networks (Callon et al., 1991) and visualization of citation networks (Chen et al., 2010). The most cited article in the cluster and second most cited in the dataset is the article introducing VosViewer (Van Eck and Waltman, 2010), a free software used for bibliometrics research popular due to its simple and versatile use. This cluster, along with cluster 1, is the youngest by the median publication year of its articles, and second-youngest by the average. And is the one with the largest proportion of articles containing the keyword scientometrics.

Cluster 7 of bibliometric theory contains foundational research of the field in terms of discovering and explaining statistical properties recurrently observed when measuring bibliographic data. Articles in this cluster study the presence of power laws in the distributions of authors, citations, and other bibliographic features (Price, 1976; Egghe, 2005b). Also, some publications target the topic of the Hirsh index (Costas and Bordons, 2007; Alonso et al., 2009). This is the oldest cluster in the network and the one with the highest concentration of articles identified by their authors as informetrics research.

Cluster 8 aggregates methods and case studies for the assessment of research and development, innovation management, academia-industry collaborations, and others. This collection of topics is referred to as tech mining or Technometrics. These articles contain bibliometric research beyond academic production, including patents, industry, and financial reports. And their methods lie in the intersection of classic bibliometrics and econometrics. Top research in this cluster includes the use of bibliometrics for defining technology roadmaps (Kostoff and Schaller, 2001; Kajikawa et al., 2008) and the use of patents and academic literature for the detection of emerging technologies (Watts and Porter, 1997; Daim et al., 2006; Rotolo et al., 2015). A prominent subtopic within this cluster corresponds to bibliometrics for the measure of interdisciplinarity in research (Porter and Rafols, 2009; Rafols and Meyer, 2010; Wagner et al., 2011). Articles in this cluster have the largest average citations received.

Clusters 9 and 10 are different in that they do not focus on a topic or research field. Instead, they are a representation of regional publication patterns. Cluster 9 contains bibliometric studies in a plurality of topics with the common characteristic that most authors of those studies are affiliated with a Spanish institution. A Spanish institution appears in the list of affiliations on 49% of the articles in the cluster, and 46% of articles have a corresponding author with a Spanish address. The second-largest country is Brazil with 8% of publications in the cluster. The 10 most mentioned institutions are also from Spain, being the University of Granada the largest contributor having an 11% of this cluster’s publications. The most cited research corresponds to a study on scientific cooperation in Europe (Narin et al., 1991) and an overview of bibliometrics written in Spanish (Bordons and Zulueta, 1999).

Cluster 10 pertains to research from and about the Global South. The predominant country is India with 33.8% of articles in the cluster having an author affiliated to an Indian institution. Follow by South African institutions featured in 10.3% of articles. Latin American countries also appear in this cluster. Besides the geographic relationship, a transversal topic is that of collaboration networks. Most cited articles include global studies on scientific collaboration (Subramanyam, 1983; Schubert et al., 1989), regional studies including the scientific collaboration network and contribution of Africa (Tijssen, 2007), or targeting specific topics like Latin-American research on AIDS (Macias-Chapula et al., 1998). Country-level bibliometrics is common like those focusing on India (Garg and Tripathi, 2018) and other countries of the Global South.

Cluster 11 contains research on Altmetrics, webometrics, scholarly communication, and intersecting studies between academia and social media. Earlier articles in the clusters attempt to understand the possibilities of using content on the Internet to replace or complement well-spread scientometrics indicators (Cronin, 2001). These articles developed their sub-field, and their applications and implications of usage are the core of this cluster. In particular, studies discussing their effectiveness (Thelwall et al., 2013) or comparability to citation-based indicators (Costas et al., 2015). One target and source of data for articles in this cluster is Twitter (Eysenbach, 2011), although several platforms are being considered within Altmetrics, including academic social networks, reference managers, blogging and microblogging, video and data sharing, wikis, ratings and review, and others (Sugimoto et al., 2017). This cluster has gained attention in the academic community, being the second cluster with the largest average number of citations received.

Although bibliometrics can be argued to be part of the field of library and information science, when studying the network of bibliometric research, it also appears as a distinctive cluster. Cluster 12 is the one collecting bibliometric studies on information and library science. Here, we find articles applying bibliometric methods to track the evolution of the field (Lariviere et al., 2012), study its topical structure (Milojevic et al., 2011), or the collaboration networks (Hou et al., 2008). Additionally, several case studies on journal-level bibliometrics appear (Anyi et al., 2009) with Scientometrics being a usual target (Schubert, 2002).

Finally, Cluster 13 collects bibliometric studies on surgery. It covers cases studies across several sub-fields of surgery. Articles covering bibliometrics on neurosurgery (Khan et al., 2014) and anesthesiology (Pagel and Hudetz, 2011) are among the top-cited. Researchers in this cluster attempt to measure the productivity in terms of the number of publications and citations received by academic surgeons to evaluate, for instance, how these metrics impact procuring competitive funding.

These 13 clusters also vary in their topical spread. For instance, clusters 9 and 10 based on regional trends cover a large variety of bibliometric-related topics. This is observed in Figure 4 with those clusters having edges spreading towards different directions in the network. Similarly for the clusters of library science and Altmetrics. Other clusters like science mapping, tech mining, and bibliometrics in surgery seem to be more cohesive, meaning that their knowledge base stays more on-topic, and their edges are less spread across the network.

**FIGURE 5:**
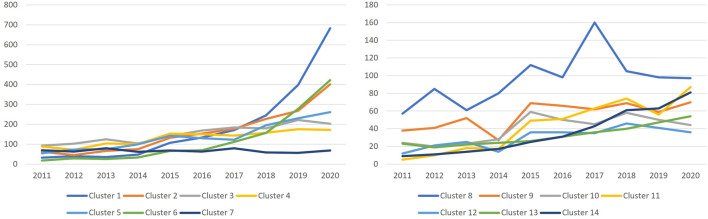
Yearly publications per cluster from 2001 to 2020.

These clusters show different publication trends over time as seen in Figure 5. Clusters of bibliometrics in management, public health, and science mapping showed the largest increase by the number of publications over the past few years. The cluster of bibliometric theory has retained a stable number of publications over the decade.

**FIGURE 6:**
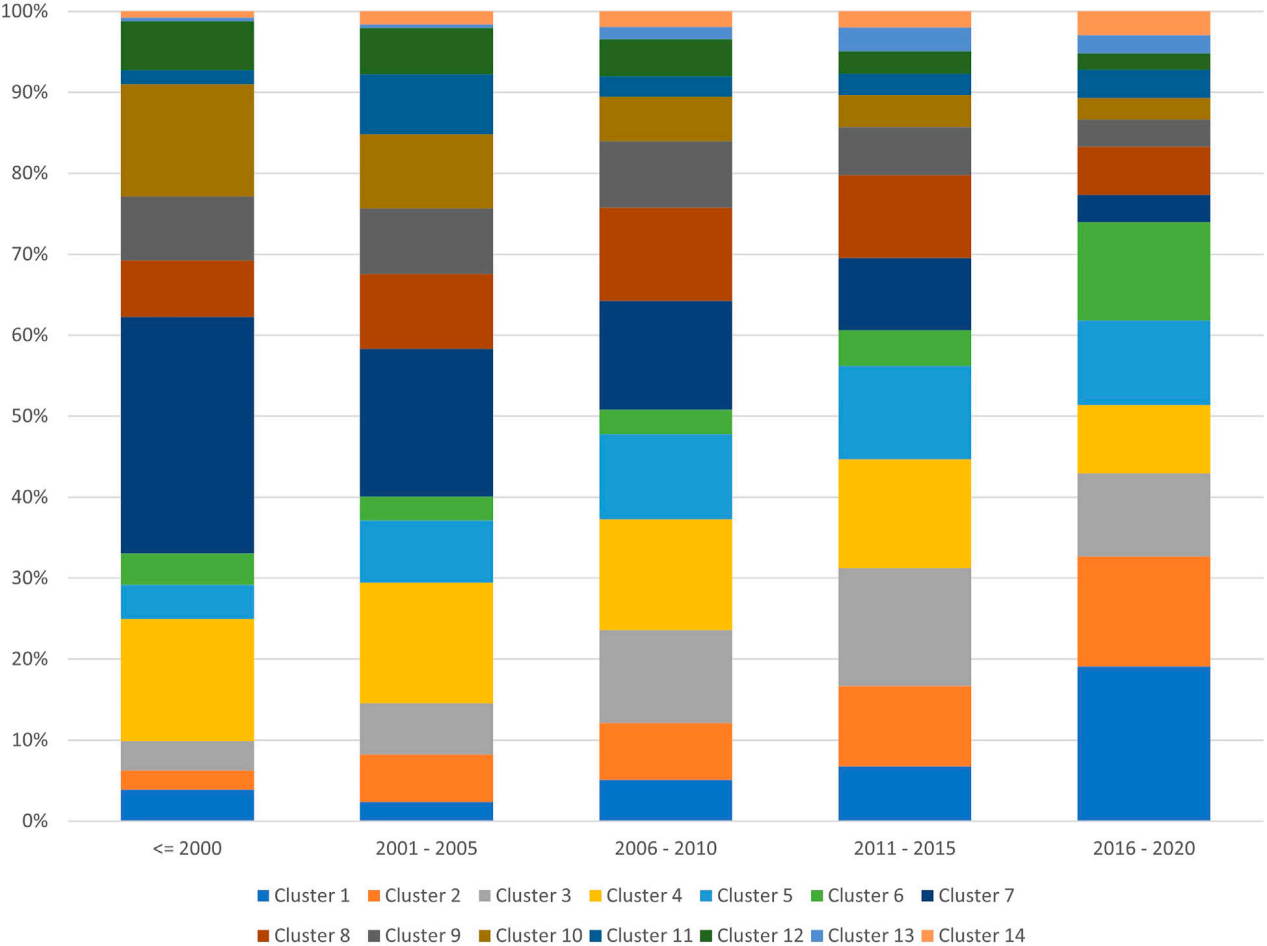
Topical cluster proportion for different windows of time until 2020.

Figure 6 shows the prominence of the clusters over different periods. The early days of bibliometrics research, considering the articles published in 2000 and before show that the dominant topic was that of bibliometric theory and research evaluation. However, in recent years their share of publications, in particular for theoretic works has decreased. This space has been taken over for cases studies in management, public health, and sustainability. Science mapping literature has seen its largest increase in the past 5 years.

### Topical Relationship Identification From Co-Term Statistics

Aiming to further investigate the relationships among scientific topics, we employed two co-term-based approaches to identify two types of relationships respectively: 1) The approach of hierarchical topic tree (HTT) for using a bird’s eye view to profile the landscape of a given scientific domain in a hierarchical structure, and 2) the approach of scientific evolutionary pathways (SEP) to track the evolution of scientific topics over time and identify their predecessor-descendant relationships from a semantic perspective.

#### Hierarchical Relationships Among Bibliometric Topics *via* Hierarchical Topic Tree

**FIGURE 7:**
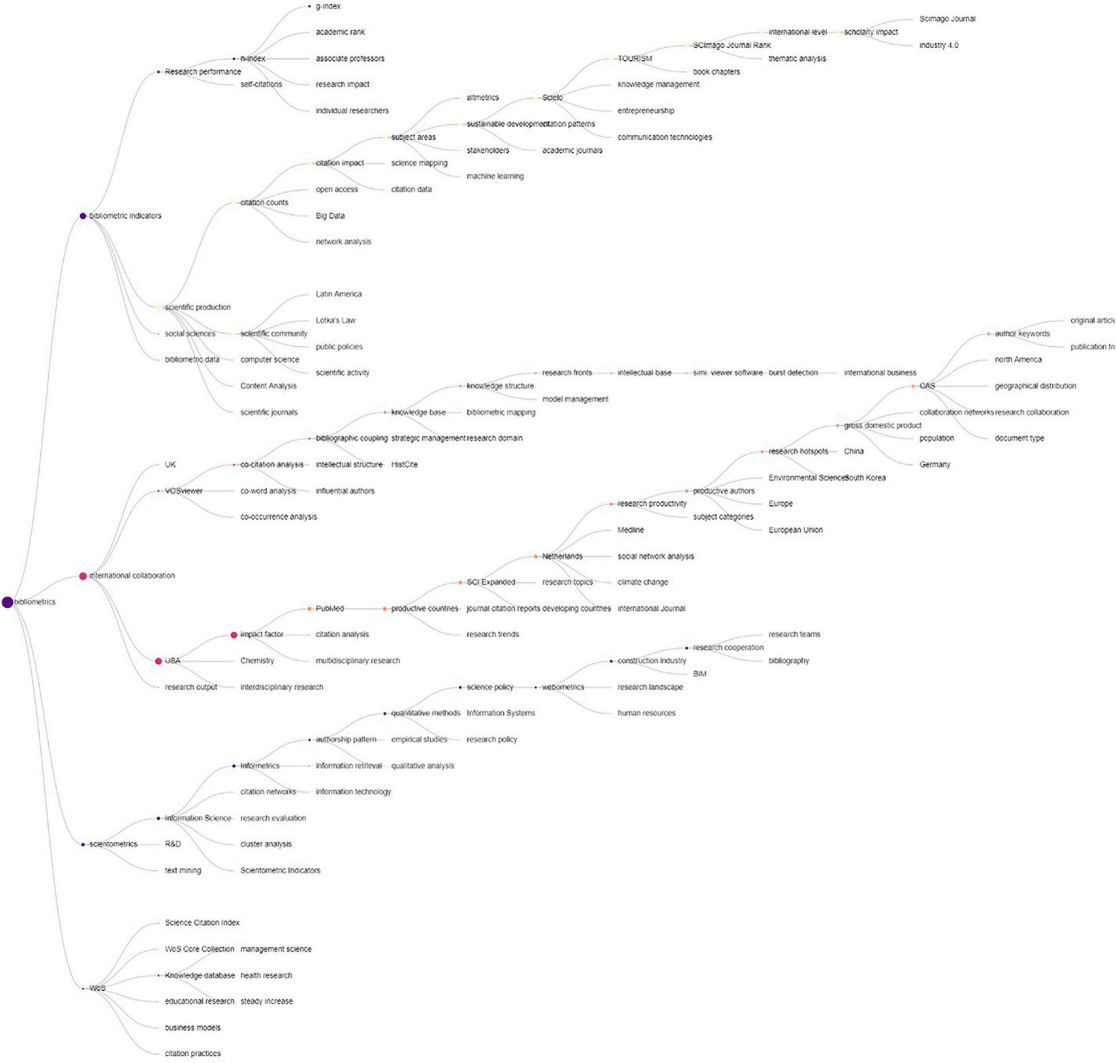
Hierarchical topic tree of bibliometric research.

The HTT of bibliometric research is given in Figure 7. Four main branches of bibliometric research are observed: 1) WoS, mainly touching the bibliometric data sources attached with WoS; 2) Scientometrics, highlighting the core of bibliometric methods and their interactions with multiple disciplines, e.g., information science, information systems, information retrieval, and policy studies; 3) International Collaboration, which mainly involves bibliometrics-based case studies, and particularly, a sub-branch labeled as VoSViewer emphasizes the use of science maps in such studies; and 4) Bibliometric Indicators, with a specific focus on research evaluation using citation indicators.

When comparing with observations identified from Figure 4, it is interesting to notice that the HTT re-organizes research clusters in bibliometrics into a relatively high granularity, and we can also easily connect the four branches in Figure 7 with the 14 clusters in Figure 4. For example, Clusters 3 “citation-based indicators” and 11 “altmetrics” are within the branch “bibliometric indicators”; and clusters related to bibliometric applications in diverse disciplines/topics such as management, public health, sustainability, and surgery, together with clusters on bibliometric studies in specific countries and regions such as Spain and Global South, can be included in the branch “international collaboration”.

#### Evolutionary Relationships Among Bibliometric Topics Between 1969 and 2020 *via* Scientific Evolutionary Pathways

**FIGURE 8:**
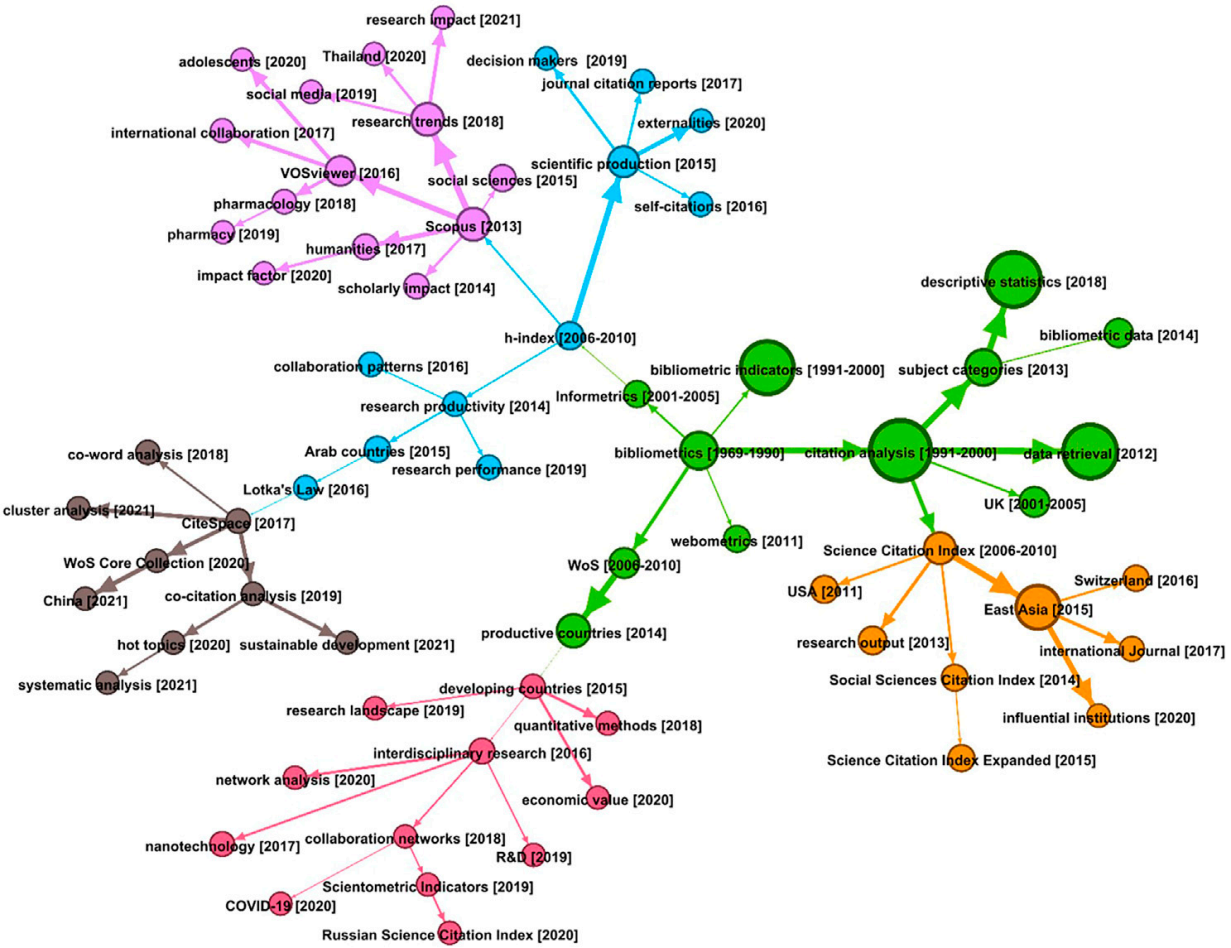
Scientific evolution pathways of bibliometric research between 1969 and 2020.

The approach of scientific evolutionary pathways (SEP) (Zhang et al., 2017) was applied to further understand these four branches of bibliometric research by tracking their evolution over time and specifically addressing the question of how the interest of the bibliometrics community was developed in the past several decades. The SEP of bibliometric research between 1969 and 2020 is given in Figure 8–a node represents a topic, the size of a node represents the number of articles involved in this topic, and the arrow between two nodes indicates their predecessor-descendant relationship, weighted by their semantic similarity.

As shown in Figure 8, the SEP identifies six research clusters, referring to the evolving interests of the bibliometrics community between 1969 and 2020. Specifically, the green cluster could be considered as the foundation of bibliometrics, consisting of bibliometric indicators (particularly citation statistics) and bibliometric data sources (e.g., WoS). Intriguingly, despite a core pillow in bibliometrics and with the largest nodes (indicating a large number of articles are still within these topics), this cluster does not evolve any new knowledge after 2018. Two young clusters evolve from the green cluster: the orange one provides certain databases collected in WoS but focuses on bibliometric studies on different countries and regions; and the pink cluster highlights certain new interests of the community, such as interdisciplinary research, network analysis, and CoVID-19-related studies in 2020.

Similarly, rooted in the topic h-index generated during the period between 2006 and 2010, the blue cluster concentrates on the use of h-index and other citation indicators in research evaluation–the other focus of the community, but the most recent topics in this cluster were generated in 2019. In terms of key interest in research evaluation, the purple cluster involves two main branches–i.e., research trends and international collaboration, in which VoSViewer was identified as a core tool. The brown cluster highlights science map-related topics, such as co-world analysis, cluster analysis, and co-citation analysis, and interestingly, raising interest in sustainable development was detected in 2021.

Another interesting topic to discuss is the consistency of the results identified from the citation networks and the co-term-based topic studies. On one hand, citations are subjectively annotated by authors, given their expertise in a specific area, while the co-term relationships only stand on the semantics, with the assumption that if two terms frequently occur together, they may share semantic similarities. Thus, citation networks may provide implicit relationships, compared to explicit information retrieved by co-term analysis. On the other hand, the citation network is based on individual research articles and citations connect articles within similar knowledge flows, but co-term analysis highlights the use of individual terms, which might cross multiple articles and provide detailed messages with relatively small granularity. Given that, Figure 4 draws a landscape of bibliometric research, and Figure 7 and Figure 8 enrich this landscape with horizontal and vertical relationships. We may consider these two sets of approaches to create complementary value for understanding bibliometric literature.

## Discussion and Conclusion

This article has explored topical representations for the academic literature acknowledged to be part of bibliometric research. To obtain these representations we have exploited the patterns found in the citation network of articles, applied advanced bibliometric methods to establish topical hierarchies, and a combination of both to surface evolutionary topical pathways.

### Key Findings

We observe that although the three terms on the study are conceptually overlapped, authors tend to label their works by choosing any of the terms. Only 8.5% of articles in the datasets show a combination of the bibliometrics, scientometrics, or informetrics terms across their title, abstract, and keywords. With bibliometrics being the prevailing one. This is also the term with more spread across the clusters, with the presence of the term having a positive correlation with the share of bibliometrics (i.e. the larger the cluster the larger the share of articles where “bibliometrics” appear). Compared to the presence of the other two terms, we observe that scientometrics and informetrics remain closer to the core of information metric research. As these have a larger share of publications on topics related to citation-based indicators, research evaluation, science mapping, and bibliometric theory.

These results are partially aligned to those of Jonkers and Derrick (2012) and González-Alcaide (2021) on the spread of bibliometric research beyond library science. However, we bring to attention an important nuance. It is “bibliometrics” as a term the one that has abruptly spread beyond the confines of information and library science. On those other disciplines, it seems that “bibliometrics” is used as a proxy to “everything statistics on publication data”, hence, the lack of recognition of foundational laws, models, and theories that concerns these authors. Scientists who identify their work as part of the corpus of “scientometrics” or “informetrics” may be more inclined to acknowledge the fundamentals.

A difference to previous studies lies in the citation impact revealed from topics in the network. Previous literature points that bibliometric studies in other fields have a marginal impact (Ellegaard and Wallin, 2015), or at least less impact (González-Alcaide, 2021) than bibliometric studies within information and library science regarding citations received. However, in our network, tech mining, a topic tightly connected to innovation, entrepreneurship, and industry collaboration has the largest average citation. Bibliometrics in management and sustainability also outperform the topics of bibliometric theory and science mapping by this metric. Therefore, citation impact varies fields to field with bibliometric studies in management and sustainability having a higher average citations than those in information and library science, while in the other end we find bibliometrics studies in public health and those from clusters of international collaboration, like in the Global South, with less citations on average.

Here, we also point out the connections to the four major branches of bibliometric indicators, international collaborations, scientometrics, and WoS (database) in the HTT as seen in Figure 7. Some research trends are identified in both citation and semantic analysis. An apparent one is the topic of research collaboration. In the citation network, the cluster of bibliometrics from Spain and the Global South correspond to patterns of citation at a regional level. Geographical proximity is known to play a role in citation patterns (Abramo et al., 2020) and related terms to international collaboration also appear in the semantic analysis. On the other hand, although related terms appear as hanging leaves in the HTT, we do not find specific branches focusing on management, sustainability, tech mining, and others, which are surfaced in the citation network clusters as shown in Table 1. This implies that other topics like indicators, collaborations, and databases are concepts that are shared by different research clusters and thus regarded as fundamentals of bibliometrics. For instance, bibliographic databases are an essential component of bibliometric research but should be integrated with other information like R&D, science policy, collaborations, economic values, and webometrics as revealed with SEP in Figure 8. From a practical perspective, we observe that the integration of multiple topical extraction methods offers an efficient and useful indicator of the evolution of the field. Further studies for the development of relevant and effective indicators are still required at different levels of topical granularity and in diverse research fields from management, health, sustainability, and others, to establish the true academic and practical impacts of bibliometrics research.

### Limitations and Future Work

The scope of the present paper is limited by the data extraction approach. We target articles where the authors recognize their work to be part of bibliometric research due to the usage of the specific terms bibliometrics, informetrics, and scientometrics. Hence, our data collection strategy was determined by our aim of capturing the academic landscape of the *usage* of those terms across all fields of science. We do not aim to say that the three terms *define* the field of bibliometrics. Defining an academic field through a search query is challenging. For instance, 61.5% of articles published in the journal *Scientometrics* in the WoS as of August 31, 2021, do not contain any of the three terms in their title, abstract, or keywords. Nevertheless, most if not all the articles in that journal can be considered to be part of the field of bibliometrics. Researchers have attempted to track the field by searching the publications of specific journals but as the field grows the selection of journals that represent a field becomes a subjective exercise. Next, we can question if a research field can even be captured by a topical search query. In information retrieval, several methods have been created for the systematic expansion of query terms for searching articles in specific topics (Carpineto and Romano, 2012). These methods rely on the existence of a ground truth dataset to optimize against, and in selecting a trade-off between precision and recall. Thus, either noise or overfitting can be expected by design. The door is open for future research to attempt developing ground truth datasets for the bibliometrics field, develop comprehensive search queries using systematic methods, and create academic landscapes based on articles pulled by those queries.

Another challenge concerns the extraction of topics from bibliometric data. We agree with the views of Gläser et al. (2017) when pointing out that as bibliometricians extracting topics from bibliographic data “we do not simply ‘discover’ the topics that ‘are in the data’ but actively construct them” based on the decisions taken in the selection of the algorithms. Hence, different solutions can be derived from the same data (Velden et al., 2017). In this research, we extracted topics from the citation network of articles, a recurrent approach in bibliometrics, but by no means the best. As a “best” approach does not exist. In this article, we brought two other representations that use advanced bibliometrics to regroup the data into hierarchical topics and evolutionary pathways. We observed from them that repeated trends exist across the methods, like those related to the theory of bibliometrics, several instances of geographic-related keywords, and a recent interest in Sustainability research. Our approach brings three different snapshots of the data using reproducible methods, but other studies bringing complementary solutions are possible and encouraged.

## Footnotes

1
https://apps.webofknowledge.com/

2
https://vega.github.io/vega/examples/tree-layout/
